# Metastatic Soft Tissue Sarcoma With Cardiac Involvement: A Case Study on the Role of Multimodal Imaging in Diagnosis and Management

**DOI:** 10.1002/ccr3.72112

**Published:** 2026-09-16

**Authors:** Azin Alizadehasl, Sina Akhavan, Seyedeh Fatemeh Hosseini Jebelli, Amirafraz fallah najmabadi, Nargeuss Abbaszade Marzbali, Fateme Yousefimoghaddam, Mehrdad Haghazali, Sajjad Rafiei

**Affiliations:** ^1^ Cardio‐Oncology Research Center, Rajaie Cardiovascular, Medical, and Research Center, School of Medicine Iran University of Medical Sciences Tehran Iran; ^2^ Cardiovascular Research Center, Rajaie Cardiovascular, Medical and Research Center Iran University of Medical Sciences Tehran Iran; ^3^ School of Medicine Shahid Beheshti University of Medical Sciences Tehran Iran; ^4^ School of Medicine Birjand University of Medical Sciences Birjand Iran

## Abstract

This case highlights the critical role of multimodal imaging in diagnosing and managing rare cardiac metastasis from soft‐tissue sarcoma, emphasizing the importance of early detection and a multidisciplinary approach to improve outcomes.

## 1 Introduction

Cardiac metastasis, a rare and unique aspect of oncology, particularly in patients with advanced soft‐tissue sarcomas, is the focus of this case study. It is a condition that occurs in only 2.3% to 18.3% of cancer patients, as per autopsy studies [1]. Soft‐tissue sarcomas, in particular, have a higher propensity to spread to the heart [2].

The symptoms of cardiac metastasis can vary, ranging from asymptomatic cases to life‐threatening conditions such as heart failure, arrhythmias, and cardiac tamponade [3]. Diagnosing and treating cardiac masses is a complex and intricate process that requires advanced imaging techniques and a high index of suspicion. Recent advancements in imaging techniques, such as cardiac magnetic resonance imaging (CMR), have significantly improved the ability to diagnose and assess the extent of cardiac involvement, adding to the complexity of the field [4].

Recent studies have shown that continued antitumor therapy can improve survival in patients with cardiac metastases [5]. This underscores the importance of a multidisciplinary approach in managing cardiac metastasis, involving cardiology, oncology, and surgical teams to provide comprehensive care. It is a testament to the necessity of collaboration in patient care [6].

This report discusses the clinical progression and imaging findings in a 36‐year‐old male with a history of soft‐tissue sarcoma, who presented with significant cardiac involvement. The case underscores the vital necessity of a comprehensive evaluation in patients with known malignancies who develop cardiac symptoms. It emphasizes the thoroughness and diligence required in patient assessment, ensuring no aspect is overlooked and highlighting the crucial role of healthcare providers in patient care [7].

## 2 Case History

A 36‐year‐old male with a known history of soft‐tissue sarcoma, diagnosed four years ago and treated with surgery, radiotherapy, and chemotherapy, presented with progressive shortness of breath and intermittent chest pain over the past few weeks. The patient, being young, was at a higher risk of developing metastatic disease. The patient had no significant cardiovascular history prior to this presentation.

After being examined, the patient was found to be stable, with a heart rate of 88 beats per minute, blood pressure of 128/76 mmHg, and oxygen saturation of 94% on room air. Cardiac auscultation revealed a soft systolic murmur best heard at the apex. There were no signs of peripheral edema or jugular venous distension. Respiratory examination revealed decreased breath sounds in the right lower lung field.

Initial investigations included an electrocardiogram (ECG) showing sinus rhythm with nonspecific ST‐T changes. A chest X‐ray demonstrated an enlarged cardiac silhouette and a right‐sided lung mass. An echocardiogram was decided upon after considering the patient's symptoms and history.

**FIGURE 1:**
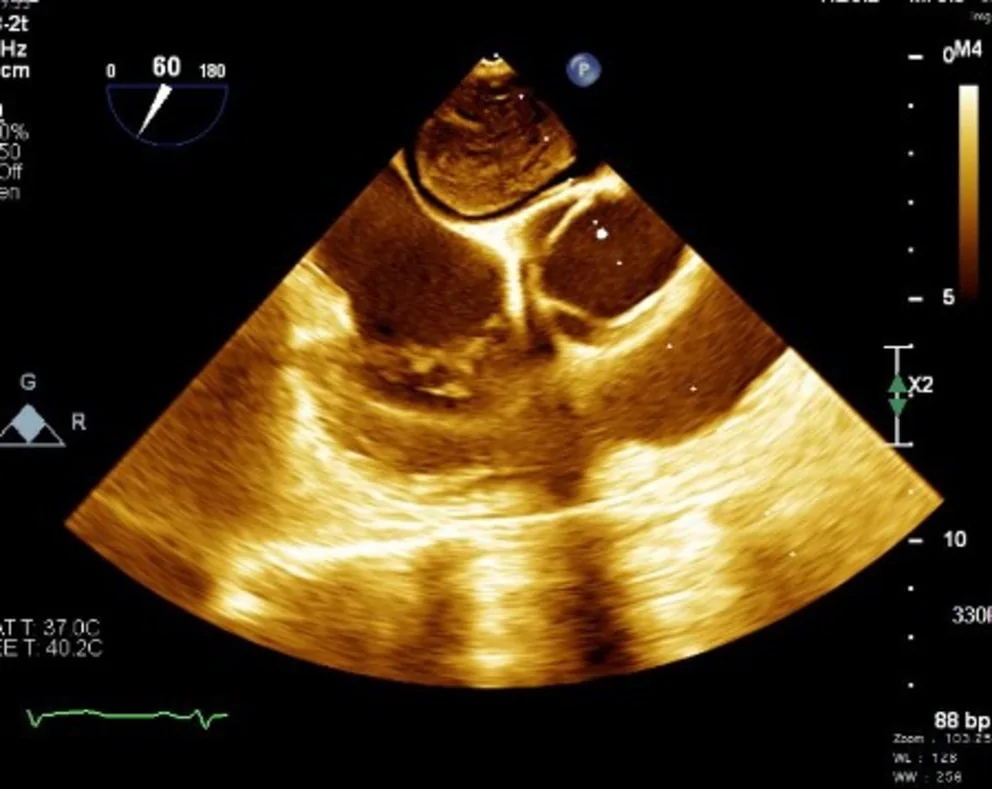
A transesophageal echocardiogram (TEE) in the short‐axis view illustrates the large (2.7 × 3.2 cm) heterogeneous mass in the left atrium, particularly impacting the right pulmonary veins.

The transthoracic echocardiogram (TTE) revealed mild‐to‐moderate left ventricle systolic dysfunction, with an ejection fraction of 40%–45%. The heart chambers and valves appeared structurally normal, except for mild mitral regurgitation (MR) and tricuspid regurgitation (TR). A large, heterogeneous mass, partially obstructing the right pulmonary veins, was notably identified in the left atrium. The mass measured approximately 5 × 3 cm, which was suggestive of metastatic involvement. (Figure 1).

Subsequent cardiac magnetic resonance imaging (CMR) further delineated the cardiac mass. The CMR confirmed a large mass (120 × 95 mm) extending from the right lung into the left atrium. The mass was heterogeneous with mild perfusion and enhancement, consistent with metastatic disease. Furthermore, the right ventricle demonstrated mildly reduced function with a proper ventricular ejection fraction (RVEF) of 43%.

A computed tomography (CT) scan of the chest was also performed, which revealed multiple pulmonary metastases, with the most significant lesion in the right lung extending into the left atrium. These findings correlated with the echocardiographic and CMR findings, indicating extensive metastatic disease involving both the lungs and heart.

Given the severity of the findings, the patient was referred to a multidisciplinary team for further management. The case was discussed in the tumor board meeting, where the decision was made to continue palliative chemotherapy and consider targeted radiation therapy for symptomatic relief, given the limited surgical options due to the extensive nature of the disease. The patient's positive response to the treatment, with reduced symptoms and improved cardiac function, as evidenced by follow‐up imaging and clinical assessments, underscores the urgency and impact of early detection and appropriate management in improving outcomes in such cases.

## 3 Differential Diagnosis & Work Up

There are several differential diagnoses for this case which consists of the following:

### 3.1 Primary Cardiac Tumors

Atrial Myxoma: The most common primary benign cardiac tumor, usually arising in the left atrium. Its heterogeneity can mimic metastasis but is usually not invasive.

Rhabdomyosarcoma: A rare primary malignant tumor that could present with similar imaging findings.

### 3.2 Metastatic Cardiac Tumors

Lung Cancer Metastasis: Commonly spreads to the heart, particularly in cases with a known primary lung tumor.

Other Soft‐Tissue Sarcomas: Known for hematogenous spread, with cardiac involvement being a rare but significant manifestation.

### 3.3 Thrombus

Intracardiac Thrombus: Often forms in patients with atrial fibrillation, heart failure, or hypercoagulable states. It can mimic a mass on imaging but lacks enhancement on CMR.

### 3.4 Infectious/Infiltrative Conditions

Infective Endocarditis: Vegetations can mimic a cardiac mass, particularly in immunocompromised patients.

Cardiac Sarcoidosis: Rare, but granulomatous infiltration can mimic malignancy.

### 3.5 Others

Lipoma or Liposarcoma: Benign or malignant fatty tumors can present similarly.

Hemangioma: Rare benign vascular tumors.

Pericardial Cyst or Pseudotumor: Uncommon but could potentially appear as a mass.

In this case, the patient's history of soft‐tissue sarcoma and the imaging findings strongly suggest metastatic involvement, as confirmed by histopathology or clinical progression. However, ruling out these differential diagnoses using multimodal imaging (echocardiography, CMR, and CT) was essential for accurate diagnosis and management.

**FIGURE 2:**
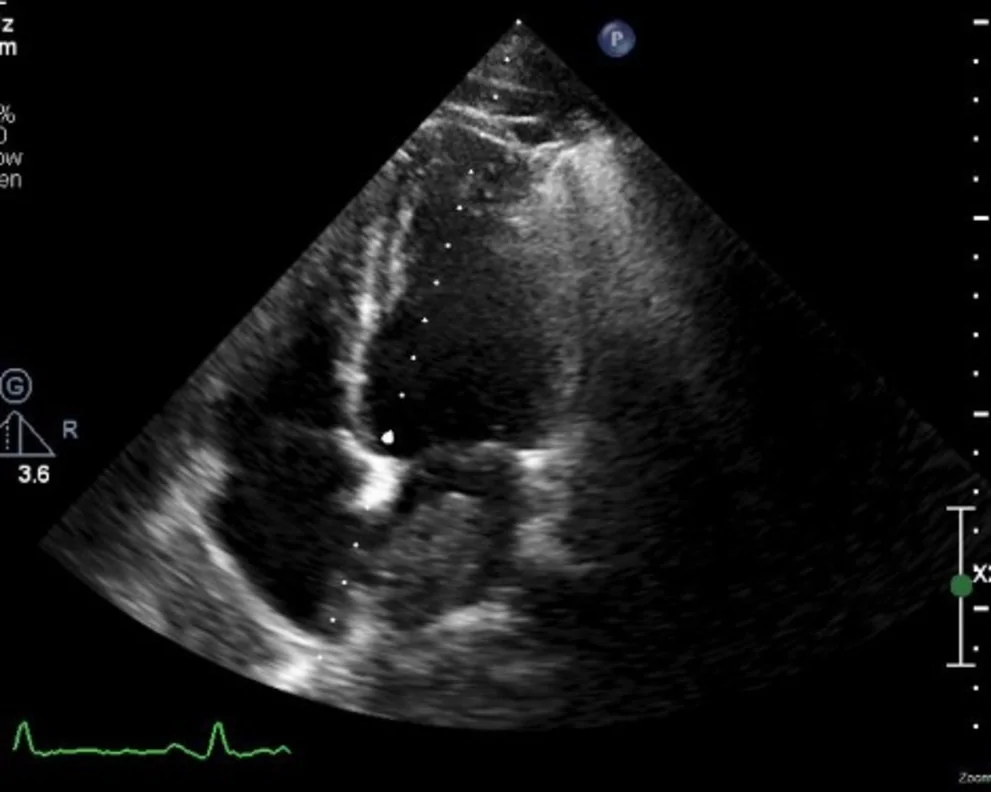
A transthoracic echocardiogram (TTE) in the 4‐chamber view, showing the left atrial mass and its significant effect on the right pulmonary veins.

The transthoracic echocardiogram (TTE) revealed several vital abnormalities. The overall cardiac structure, including the size and configuration of the heart chambers and valves, appeared normal, which is significant in ruling out primary structural heart disease (Figure 2). However, the systolic function of the left ventricle (LV) was compromised, showing a mild‐to‐moderate reduction in ejection fraction, estimated between 40% and 45%. This reduction suggests a decreased pumping ability of the heart, potentially contributing to the patient's symptoms of shortness of breath and chest discomfort.

The echocardiogram also identified mild mitral regurgitation (MR) and tricuspid regurgitation (TR), indicating minor blood backflow through the mitral and tricuspid valves during systole. Moreover, pulmonary artery pressure was slightly increased, possibly in response to the underlying pulmonary pathology or as a secondary effect of left‐sided heart issues.

The most significant finding was a large, heterogeneous mass measuring approximately 2.7 × 3.2 cm within the left atrium. This mass intruded on the right pulmonary veins, which could explain the observed hemodynamic effects, including obstructing pulmonary venous return, thereby exacerbating left ventricular dysfunction by decreasing ventricular filling.

**FIGURE 3:**
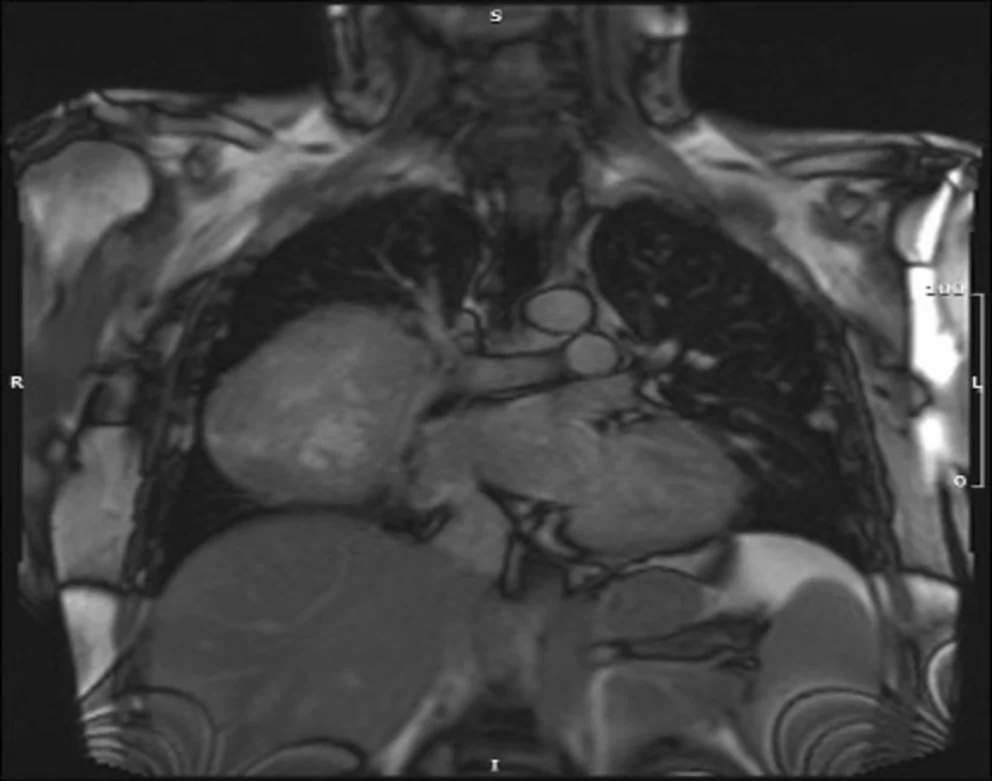
4‐Chamber Long Axis SSFP Image.

**FIGURE 4:**
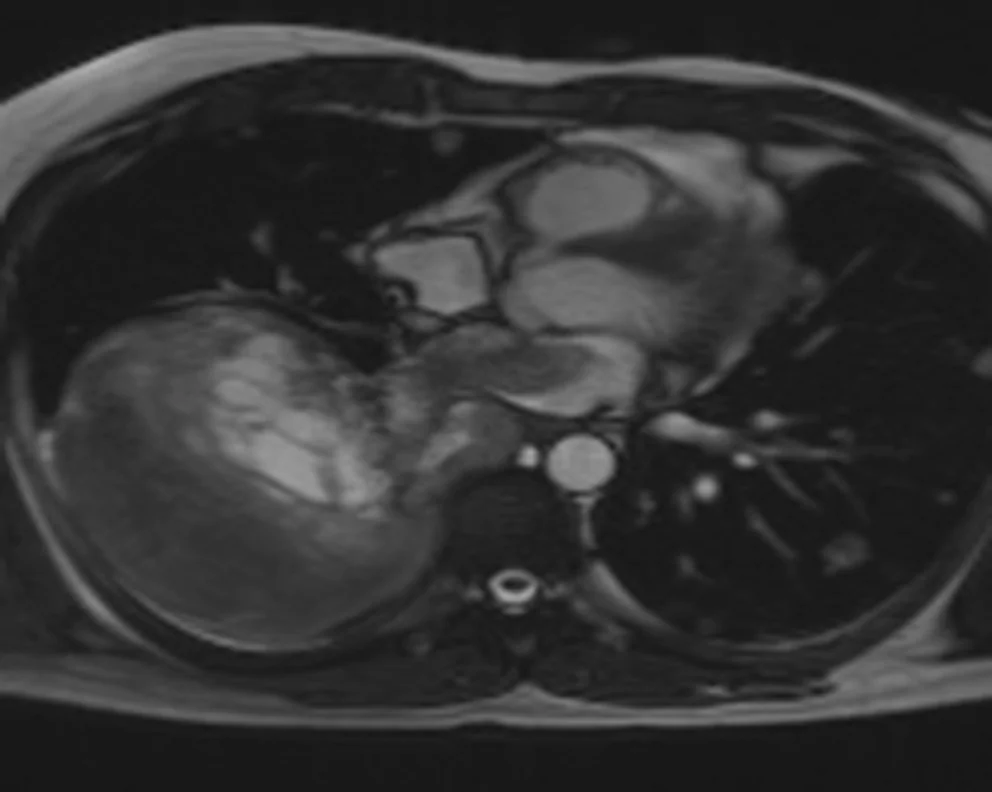
Axial SSFP Image of a Large Heterogeneous Right Lung Mass Infiltrating the Left Atrium via the Pulmonary Vein with Metastatic Nodules in the Left Lung.

**FIGURE 5:**
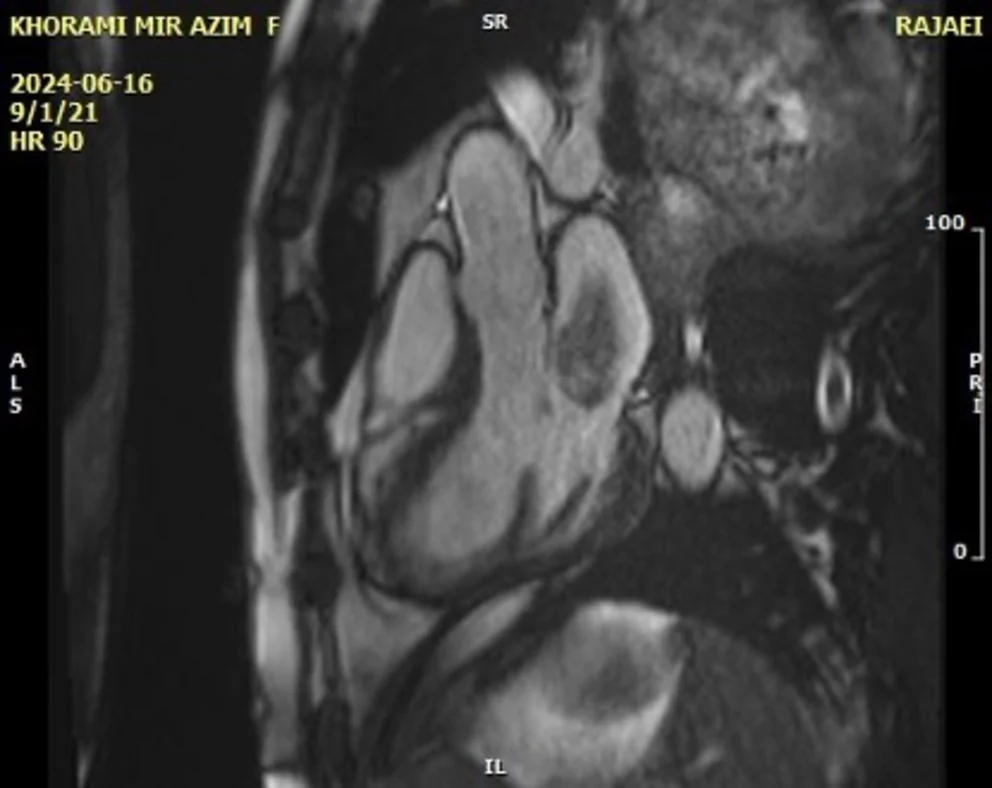
3‐Chamber Long Axis SSFP Image Revealing Heterogeneous Signal of Mass Lesion in the Left Atrium.

Cardiac Magnetic Resonance (CMR) Findings:

Cardiac magnetic resonance imaging (CMR) was conducted to evaluate the cardiac mass further and provide more detailed anatomical and functional insights (Figure 3). The CMR confirmed the presence of a large mass extending from the right lung into the left atrium, consistent with metastatic disease from the patient's known primary soft tissue sarcoma. The mass measured 120 × 95 mm and was characterized by heterogeneous tissue with mild perfusion and enhancement, typical of metastatic tumors (Figures 4, 5).

The right ventricle (RV) showed a mild reduction in function, with a proper ventricular ejection fraction (RVEF) of 43%. Despite the reduced function, the RV maintained a standard size, which is crucial for comprehending the extent of the cardiac involvement and the impact on the heart's ability to compensate for the tumor's presence.

In addition, the CMR showed minimal aortic insufficiency (trivial AI), a minor finding relevant to comprehensive cardiac evaluation. There was also evidence of localized increased T1 signal in the myocardium, suggesting possible chemotherapy‐induced cardiomyopathy, a common adverse effect in patients with a history of chemotherapy for malignancies.

The Computed Tomography (CT) findings:

A computed tomography (CT) scan of the chest was performed to assess the extent of metastatic disease. The scan revealed multiple pulmonary metastases, with the most significant lesion being in the right lung, which extended into the left atrium. This finding correlates with the echocardiographic and CMR results, providing a comprehensive picture of the extensive metastatic involvement affecting the lungs and the heart.

**FIGURE 6:**
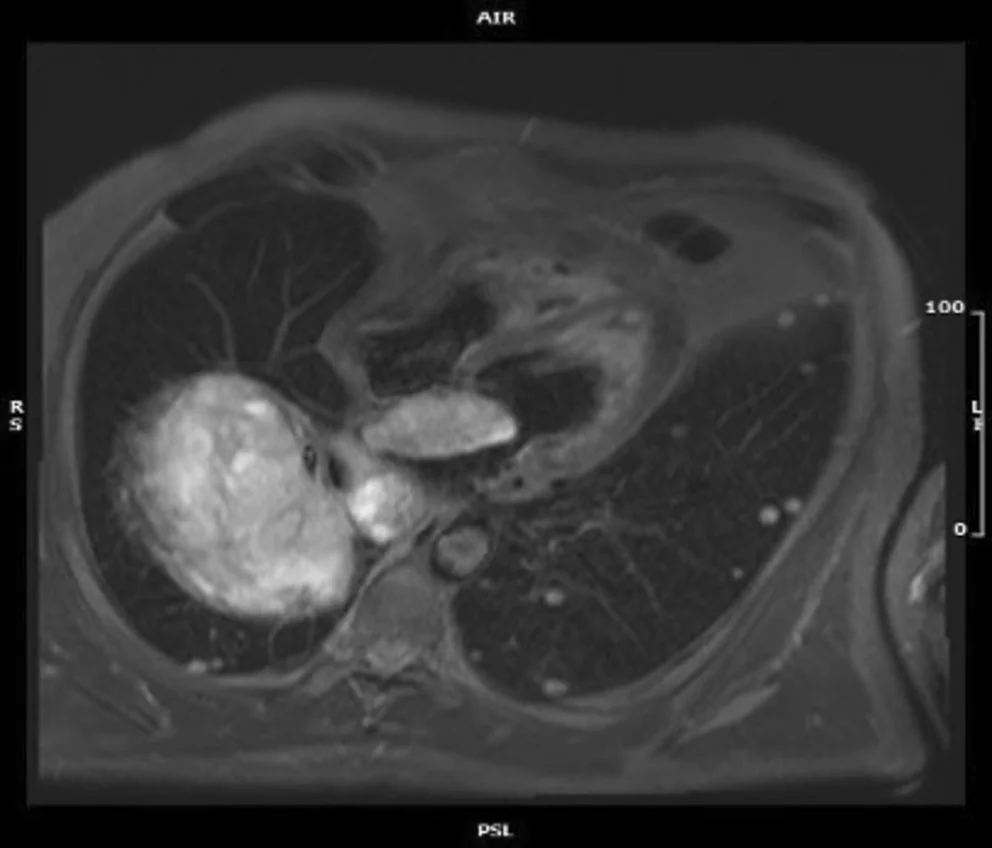
Axial STIR Image Highlighting a High‐Signal Mass Lesion in the Right Lung and Left Atrium with Multiple Pulmonary Metastases in the Left Lung.

The diagnostic workup, including echocardiography, CMR, and CT, conclusively identified a large metastatic mass in the left atrium, with associated mild‐to‐moderate left ventricular dysfunction. These findings are consistent with advanced soft‐tissue sarcoma complicated by metastasis to the heart and lungs (Figure 6). Being the cardiac mass, along with reduced cardiac function and pulmonary metastases, underscores the aggressive nature of the disease and the critical need for multidisciplinary management.

Integrating various imaging modalities was pivotal in establishing the diagnosis, revealing the extent of the disease, and guiding therapeutic decision‐making. This case highlights prizing thorough and timely cardiac evaluation in patients with known malignancies, those with primary tumors prone to cardiac involvement.

## 4 Conclusion and Results

Cardiac metastasis, a rare and severe complication of advanced malignancies, often presents significant diagnostic and therapeutic challenges. This case report brings to light the unique and intricate relationship between oncological progression and cardiac involvement, using the example of a 36‐year‐old male with a history of soft‐tissue sarcoma who developed significant cardiac metastasis leading to symptomatic heart failure.

The rarity of cardiac metastasis, particularly from soft‐tissue sarcomas, accentuates the diagnostic dilemma clinicians face. Cardiac metastases often remain silent until they reach a critical size or location, causing symptoms that can be mistaken for primary cardiac conditions. The patient's presentation with progressive dyspnea and intermittent chest pain led to an extensive cardiovascular evaluation. After identifying a large mass in the left atrium, which obstructed the right pulmonary veins, it was underscored to consider metastatic disease in patients who had a known history of malignancy.

Advanced imaging, particularly multimodal imaging, was pivotal in this patient's accurate diagnosis and management planning. The mass was initially discovered through transthoracic echocardiography (TTE), with its metastatic nature subsequently confirmed by cardiac magnetic resonance imaging (CMR). The findings of mild‐to‐moderate left ventricular dysfunction and the mass interference with pulmonary venous return were critical in understanding the extent of cardiac involvement and the resultant hemodynamic impact, underscoring the importance of technology in such complex scenarios.

The interdisciplinary approach to managing this case—incorporating oncologists, cardiologists, and radiologists—reflects the complexity of treating cardiac metastasis. Considering the extensive nature of the disease and the limited surgical options, it was both pragmatic and patient‐centered to opt for palliative chemotherapy and targeted radiation therapy. The patient experienced reduced dyspnea and chest pain, and his cardiac function stabilized following treatment, highlighting the potential benefits of timely and coordinated care.

This case underscores the urgent need for early diagnosis of cardiac metastasis in patients with known primary tumors prone to such spread. It also illustrates the indispensable role of advanced imaging in diagnosing and guiding the therapeutic approach in such complex scenarios. Early recognition and a multidisciplinary management strategy are essential in improving clinical outcomes, even in extensive metastatic disease.

## 5 Discussion

This case illustrates the critical role of cardiac imaging in detecting and characterizing metastatic disease in oncology patients. The findings highlight the aggressive nature of soft‐tissue sarcoma and its potential to spread to the heart, leading to significant cardiac morbidity. It also underscores the urgency and responsibility in early detection and management, which is crucial to improving outcomes in such cases.

## Author Contributions

**Azin Alizadehasl:** conceptualization, writing – review and editing. **Sina Akhavan:** writing – original draft. **Seyedeh Fatemeh Hosseini Jebelli:** writing – original draft. **Amirafraz fallah najmabadi:** writing – original draft. **Nargeuss Abbaszade Marzbali:** writing – review and editing. **Fateme Yousefimoghaddam:** conceptualization, writing – original draft. **Mehrdad Haghazali:** conceptualization, writing – original draft. **Sajjad Rafiei:** conceptualization, writing – original draft.

## Funding

The authors have not declared a specific grant for this research from any funding agency in the public, commercial, or not‐for‐profit sectors.

## Consent

Written informed consent was obtained from the patient's next of kin to publish this report in accordance with the journal's patient consent policy.

## Conflicts of Interest

The authors declare no conflicts of interest.

## Data Availability

The data that support the findings of this study are available from the corresponding author upon reasonable request.

## References

[1] A. C. Gamboa , A. Gronchi , and K. L. Cardona , “Soft‐Tissue Sarcoma in Adults: An Update on the Current State of Histotype‐Specific Management in an Era of Personalized Medicine,” CA: A Cancer Journal for Clinicians 70, no. 3 (2020): 200–229, 10.3322/caac.21605. 32275330

[2] S. Li , Q. Sun , R. Bai , et al., “Real‐World Efficacy, Safety Data, and Predictive Clinical Parameters for Treatment Outcomes in Advanced Soft Tissue Sarcoma Treated With Combined Immunotherapy and Antimicrobial Therapy,” BMC Cancer 24 (2024): 1028, 10.1186/s12885-024-12810-9. 39164643 PMC11337792

[3] C. S. Rutland , “Advances in Soft Tissue and Bone Sarcoma,” Cancers 16, no. 16 (2024): 2875, 10.3390/cancers16162875. 39199646 PMC11352553

[4] Z. Lin , H. Xiao , J. Liu , et al., “Clinical Features and Prognosis of Cardiac Metastatic Tumors,” BMC Cancer 23 (2023): 1235, 10.1186/s12885-023-11733-1. 38102550 PMC10722672

[5] A. Al‐Mamgani , L. Baartman , M. Baaijens , I. de Pree , L. Incrocci , and P. C. Levendag , “Cardiac Metastases,” International Journal of Clinical Oncology 13 (2008): 369–372, 10.1007/s10147-007-0749-8. 18704641

[6] H. Sarfraz , A. Arain , M. K. Divatia , M. R. Schwartz , and K. E. Heyne , “Metastatic Colorectal Carcinoma to the Right Atrium: A Case Report and Review of the Literature,” Cardio‐Oncology 7 (2021): 21, 10.1186/s40959-021-00108-9. 34059136 PMC8165980

[7] R. Bussani , M. Castrichini , L. Restivo , et al., “Cardiac Tumors: Diagnosis, Prognosis, and Treatment,” Current Cardiology Reports 22 (2020): 169, 10.1007/s11886-020-01420-z. 33040219 PMC7547967

